# How the “Are We Drinking Ourselves Sick?” Communication Campaign Built Support for Policy Action on Sugary Drinks in Jamaica

**DOI:** 10.3390/nu14142866

**Published:** 2022-07-13

**Authors:** Donnelle Christian, Meena Maharjan, Alexey Kotov, Trish Cotter, Sandra Mullin, Vonetta Nurse, Barbara McGaw, Deborah Chen, Pallavi Puri, Shuo Wang, Nalin Singh Negi, Nandita Murukutla

**Affiliations:** 1 Policy Advocacy and Communication Division, Vital Strategies, New York, NY 10005, USA; dchristian@vitalstrategies.org (D.C.); akotov@vitalstrategies.org (A.K.); tcotter@vitalstrategies.org (T.C.); smullin@vitalstrategies.org (S.M.); ppuri@vitalstrategies.org (P.P.); shuo89510@gmail.com (S.W.); nnegi@vitalstrategies.org (N.S.N.); nmurukutla@vitalstrategies.org (N.M.); 2 Global Health Advocacy Project, The Heart Foundation of Jamaica, 28 Beechwood Avenue P.O. Box 338, Kingston 5, Jamaica; vonetta.alexis@gmail.com (V.N.); pmjctc@heartfoundationja.org (B.M.); execdir@heartfoundationja.org (D.C.)

**Keywords:** sugary drinks, Jamaica, mass media campaign, tax

## Abstract

Background: This study assesses the effectiveness of a campaign “Are We Drinking Ourselves Sick?” that ran nationally in Jamaica in four phases from 2017 to 2019 to increase knowledge about the harms of sugary drinks, shift attitudes, and build support for policy actions to address sugary drink consumption, including a tax and a ban in schools. Methods: Campaign impact was measured in representative cross-sectional household surveys of adults ages 18 to 55. A baseline survey was conducted before the launch of the campaign (*n* = 1430). Evaluation surveys were conducted mid-campaign (*n* = 1571) and post-campaign (*n* = 1500). Campaign impact was assessed by comparing changes across survey periods on key knowledge, attitudinal and policy support outcome indicators. The independent association between campaign awareness and outcomes was analyzed using logistic regression analyses. Results: The campaign was recalled by more than 80% of respondents and was well-received with 90% or more respondents describing it as believable and relevant. There was a decline in knowledge on the harms of sugary drinks from the baseline to post-campaign period, notably on risks of diabetes (adjusted odds ratio or AOR = 0.62, *p* < 0.001), overweight and obesity (AOR = 0.58, *p* < 0.001), and heart disease (AOR = 0.79, *p* < 0.003). However, post-campaign awareness was independently associated in logistic regression analysis with improved knowledge of the harms of sugary drinks, including risks of diabetes (AOR = 1.45, *p* = 0.019), overweight or obesity (AOR = 1.65, *p* = 0.001), and heart disease (AOR = 1.44, *p* = 0.011). Support for government action remained high across survey waves (≥90%), and campaign awareness was independently associated with increased policy support for sugary drinks taxes (Mid-campaign: AOR = 1.43, *p* = 0.019; post-campaign: AOR = 1.46, *p* = 0.01) and restrictions on sugary drinks in schools (AOR = 1.55, *p* = 0.01). Conclusion: This study demonstrates the role that media campaigns can play in maintaining knowledge and concern about the health harms of sugary drinks and increasing support for policy passage.

## 1. Introduction

Sugary drinks including carbonated soft drinks, sports drinks, and energy drinks are one of the largest sources of added sugar in diets [1,2] and are a major contributor to overweight and obesity—well-established risk factors for noncommunicable diseases (NCDs) including diabetes and heart disease [3,4,5,6]. Currently, more than half of Jamaicans ages 15 and older (54%) are overweight or obese and rates are rising among young people [7,8,9], alongside the prevalence of diet-related diseases [10,11]. Therefore, targeting sugary drink consumption is critical for obesity and NCD prevention in Jamaica.

Although there are a number of evidence-based policies to improve food environments and denormalize the purchase and consumption of unhealthy drinks and foods, policymaker support and government uptake of these policies varies [12,13]. Jamaica does not currently have a comprehensive policy to restrict or ban the advertising of high-sugar foods and beverages to children on media channels. This means there are no limitations to exposure of the population—particularly children—to highly marketed sugary drinks at low prices, which are available ubiquitously across the country [14]. Prior to January 2019 when the government implemented a progressive restriction [15,16] on sugary drinks in schools, if the total sugar concentration exceeded a prescribed amount per milliliter, there were no restrictions on the direct marketing of sugary drinks in schools. Consequently, children had easy access to sugary drinks provided at school canteens or by onsite vendors [17].

Barriers to government uptake of policies to improve food environments and diets include aggressive lobbying by the food and beverage industry, [18,19] as well as unknown or perceived lack of public support, particularly for policies that have proven to be more contentious such as restrictions on product availability or financial disincentives [12,18,20,21,22]. Sugary drink taxes are an effective policy to reduce sugar consumption but have been notoriously hard to pass for these reasons [23,24,25]. Several countries in the Latin America and Caribbean region, including Barbados, Mexico, and Chile, have successfully introduced taxes on sugary drinks. Evidence from these countries supports the health, financial, and social benefits of the policy [26,27,28,29,30,31]. Prohibiting the sale or provision of sugary drinks in schools reduces in-school access [32], whereas higher taxes effectively limit young peoples’ access to sugary drinks outside of school and reduce their consumption [33]. Thus, these interventions make an effective policy package to reduce young people’s access to and consumption of sugary drinks.

Mass media campaigns have traditionally been used to positively change health behaviors and shift social norms [34,35], but are also increasingly being recognized as a tool to advance public health policies. Campaigns have been found to effectively shape public opinion on health issues, prompt discussion and channel increased concern into pressure for government action [36,37,38,39]. Evidence on mass media campaigns addressing taxes on sugary drinks shows that campaigns have shifted media narratives and public attitudes toward favoring the policy [40,41,42]. Thus, research suggests that media campaigns can serve both as a tool for advocates to set the public health agenda and for governments as they look to enact proposed policies.

Based on this evidence and in response to the need for policy-level intervention on sugary drinks, a consortium of civil society organizations led by the Heart Foundation of Jamaica with the support of Vital Strategies, implemented a mass media campaign from 2017 to 2019 to build the case for policy action. This study assesses the effect of the campaign on knowledge, attitudes, and support for public policies to address sugary drink consumption, which were defined as drinks that are naturally sugary or have sugars/sweeteners added to them (e.g., sodas, sports drinks, flavored milks, and fruit juices—excluding diet drinks). To our knowledge, it is the first to do so in Jamaica or any other Caribbean country. This study also adds to the budding literature on how mass media campaigns can build and foster support for healthy food policies, particularly those perceived to be more politically challenging.

## 2. Materials and Methods

### 2.1. Jamaica’s “Are We Drinking Ourselves Sick?” Campaign, Phases 1–4

In 2017, the Heart Foundation of Jamaica, an organization devoted to the prevention and control of cardiovascular disease and its risk factors, with the support of Vital Strategies, a global public health organization, developed a strategic mass media communication campaign, “Are We Drinking Ourselves Sick?” to build support for government action on sugary drinks. After conducting message testing, materials for traditional and digital media channels were developed and launched.

The “Are We Drinking Ourselves Sick?” campaign targeted adults 18–65 with messaging that intended to (1) increase knowledge about the health harms of sugary drinks, particularly for children, (2) shift attitudes toward sugary drinks, (3) strengthen support for government action, including a tax on sugary drinks and a healthy school nutrition policy banning sugary drinks. The campaign was launched in four sequential phases, with different ads rolled out in each phase (see Table 1). The target audience varied slightly for each phase.

**Table 1:** Campaign details with key messages.

Phase	Target Audience	Key Messages	Media Used	Name of TV ad and Image	Description
I 17 November–16 December 2017 and 8 January–9 February 2018	Women, adults 18–65 years	Drinking sugary drinks increases the risk of type 2 diabetes, heart disease, cancers.	TV, radio, newspapers, digital, outdoors.	“Journey” 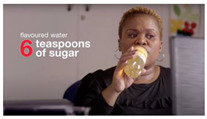	The ad follows a woman as she drinks sugary drinks throughout the day and explains how the sugar content in each drink accumulates (e.g., energy drink = 14 teaspoons of sugar). The woman is shown in the hospital suffering from heart disease, as it is explained how sugary drinks bring on obesity, which can lead to Type 2 diabetes, heart disease and some cancers. This campaign was inspired by the “Are You Drinking Yourself Sick?” campaign, developed by Healthy Living Alliance in South Africa. See Figure 1 for an example of additional campaign material launched during this phase.
II 13 February–31 May 2018	Parents and guardians with children under 16 years	Sugary drinks are a main reason for the obesity crisis in Jamaica; just because you’re active, it doesn’t mean sugary drinks aren’t causing harm to your health.	TV, radio, newspapers, digital, outdoors.	“Skinny Dad” 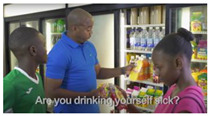	The ad shows an active family entering a gas station mini mart to purchase drinks. After considering the health consequences of the sugary drinks they’ve selected, the father opts for water for his family instead.
III 11 October–9 December 2018	Adults 18–65, parents, and guardians with children under 16 years, policymakers	All that sugar adds up; Sugary drinks can lead to Type 2 diabetes, destroy your children’s teeth by causing painful tooth decay; cut out those sugary drinks at home and at school.	TV, digital, radio, outdoors, newspapers.	“Tooth Decay” 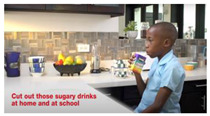	The ad depicts a mother pouring a sugary drink for her toddler, who is shown consuming similar drinks as he grows into a young man and adult. He is shown developing Type 2 diabetes and tooth decay, as a voiceover encourages that sugary drinks be cut out at home and school. This campaign was inspired by “Tooth Decay, Play Every Day” campaign, developed by the Department of Health and Social Services, State of Alaska.
IV 22 February–31 March 2019	Adults 18–65, policymakers	Support our children’s health, support a sugary drinks tax.	TV, digital, radio, newspapers, outdoors.	“Are We Drinking Ourselves Sick? Testimonies-Dentist” 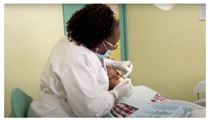	This series of ads features two testimonials from medical professionals who share the ways in which they have seen children’s health compromised by sugary drinks. A third testimonial is from a man who shares his experience with Type 2 diabetes and his hopes that what happened to him does not happen to his children. Viewers are encouraged to support children’s health by backing a sugary drinks tax. See Figure 2 for an example of additional campaign material launched during this phase.
			TV, digital, radio, newspapers.	“Are We Drinking Ourselves Sick? Testimonies-General Practitioner” 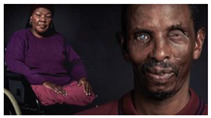	
			TV, social media, radio, newspapers.	“Are We Drinking Ourselves Sick? Testimonies- Patient” 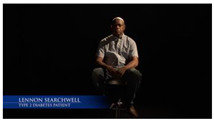	

**Figure 3:**
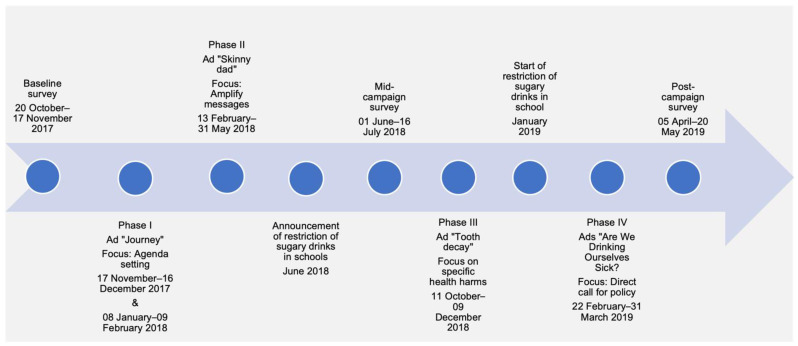
“Are We Drinking Ourselves Sick?” Communication Campaign Timeline.

To evaluate campaign effectiveness a series of cross-sectional nationally representative surveys were conducted before, between, and after the campaign phases (see Figure 3): the pre-campaign survey (hereafter referred to as “baseline survey”) was conducted before the launch of the campaign from 20 October to 17 November 2017; the mid-campaign survey was conducted after campaign phases I and II from 1 June to 16 July 2018; the post-campaign survey was conducted following the conclusion of phases III and IV from 5 April to 20 May 2019.

### 2.2. Sample and Data Collection

The three nationally representative face-to-face household surveys were conducted by the market research company, HOPE Caribbean Company Limited, under Vital Strategies’ guidance. A stratified multistage random sample design with quotas for age, gender, socioeconomic status, and location (urban/rural) from each stratum (parish, constituencies, areas, household) was used. The country was stratified into 14 parishes, which were further stratified into constituencies and then into two areas within each constituency (parish capitals and main town, and special areas using the definition of Statistical Institute of Jamaica). Each of the areas comprising the constituencies was then divided into primary sampling units or enumeration districts. A random sample of primary sampling units was then selected with a probability proportional to size, and the households within each selected enumeration district or primary sampling unit were identified using a specially designed listing form. A systematic sample of households was then selected, and one person within each household was interviewed. If more than one person in a household qualified, the last birthday method was used to select participants. Respondent eligibility included being between the ages of 18 and 55 years and not being employed by tobacco, junk food, sugary drinks, advertising, or market research companies. The samples were as follows: 1430 respondents for the baseline survey; 1571 for the mid-campaign survey; and 1500 for the post-campaign survey.

#### Ethical Approval

Prior to participation, the study was explained to participants and informed consent was obtained. The questionnaire was administered only to participants who agreed to participate. HOPE Caribbean complied with the professional and ethical standards of the Advisory Panel on Ethics and Medico-legal Affairs, Ministry of Health, Jamaica, to conduct this research.

### 2.3. Questionnaire and Measures

The structured questionnaire for all three survey waves was interviewer-administered face-to-face in English via computer-assisted personal interviewing (CAPI). The questionnaire was developed by the Vital Strategies research team based on a literature review and advice from public health experts. Pilot testing was conducted to test for accuracy, fluidity, and ease of comprehension. The core study measures were kept consistent across survey waves. However, to accommodate the slight variations in messages between campaign phases while maintaining the overall survey length, some specific items were deleted or added in the subsequent survey waves. The key measures that were used in the questionnaire are described below. This paper includes and describes in detail only the survey items that are pertinent to the evaluation. Therefore, the description below does not follow the same order of questions in the questionnaire. Additional questions in the survey, which were intended to support related food policy activities but were not directly pertinent to this evaluation, are not described in this paper but are available in the full questionnaire, available upon request.

#### 2.3.1. Campaign Awareness

Campaign awareness was measured by determining whether respondents recognized key images from the campaign ads. Respondents who recalled either of the ads in each campaign phase from any media channel were considered to have “campaign awareness” of the respective campaign phase. Those who recalled phase I and phase II campaign ads were considered campaign aware in the mid-campaign survey; those who recalled phase III and phase IV campaign ads were coded as campaign aware in the post-campaign survey; all others were coded as unaware in each respective survey wave.

#### 2.3.2. Reactions to the Campaigns

Reactions to the campaign were assessed by asking campaign-aware respondents to rate how strongly they agreed or disagreed with statements about the ads, including whether it was (1) believable; (2) relevant; (3) taught something new; (4) created concern about the health harms of sugary drinks; and (5) increased support for government action to reduce sugary drink consumption. Each statement was measured separately using a five-point Likert scale.

#### 2.3.3. Interpersonal Communication

The degree to which the campaigns spurred interpersonal communication was assessed by asking campaign-aware respondents if they had discussed the relevant ad with people in their network and if so, with whom (friends, family, colleagues, or others).

#### 2.3.4. Knowledge and Attitudes

A five-point Likert scale was used to gauge knowledge on the health harms of sugary drink consumption and on risk factors for overweight/obesity. The same scale was used to access attitudes toward sugary drink consumption and opinions on factors that may be causing an increase in consumption, including pricing and marketing.

#### 2.3.5. Support for Policies and Government Action

Support for policies and government action was assessed by asking respondents to rate a series of statements on government efforts to reduce the consumption of sugary drinks and the prevalence of overweight and obesity. Respondents indicated how strongly they agreed or disagreed with the statements, which were measured using a five-point Likert scale. Questions focused on the extent to which respondents thought the government should take action to address obesity and discourage the consumption of sugary drinks and unhealthy foods. Respondents were also asked questions to assess the degree to which they would support different types of tax policies to help address obesity in Jamaica and policies to address the consumption of sugary drinks and unhealthy foods in schools.

#### 2.3.6. Sociodemographic Characteristics

The screener questionnaire also measured sociodemographic characteristics, including gender, age, parent/primary caregiver status (to children under the age of 16), level of education, employment status, and socioeconomic status. Socioeconomic status was calculated using a proxy measure by assigning weight to education level and occupation. Additional questions related to nutrition and physical activity, health status, and media consumption were asked. These included: fruit and vegetable intake in the last week; frequency of vigorous physical activity in the last week; body mass index; self-reported health status; and frequency of media use, including television (TV), radio programming, newspapers/periodicals, and the internet.

### 2.4. Data Analysis

Data were analyzed using IBM SPSS version 25. Two sets of comparisons were made: first, comparisons by survey waves to measure changes in key outcomes over time; second, comparisons by campaign awareness to measure campaign-attributable effects on the key outcomes. In both cases, bivariate analyses were run first, followed by multivariate binary logistic regressions. The bivariate analyses entailed the use of chi-square tests for categorical variables and t-tests for categorical and continuous variables. In addition, a column proportion test (z-test) was used to determine whether there was a relationship between a categorical variable in columns and a continuous variable in the rows. This was followed by a multivariate binary logistic regression that examined changes over time, and the independent association between campaign awareness and key outcomes, while holding potential confounders constant. The campaign period (baseline, mid-campaign, and post-campaign) was regressed on dichotomized outcome measures of knowledge and attitudes toward obesity and sugary drinks and support for government efforts, including a comparison of responses from respondents who were aware and unaware of the campaign. Outcome variables were dichotomized for the analysis and adjusted odds ratios (AOR) were calculated and presented. Covariates included age, gender, parental status, education level, employment status, socioeconomic status, fruits and vegetables intake in the last seven days, vigorous physical activity in the last seven days, body mass index, self-reported health status, and frequency of watching TV, listening to the radio, reading newspapers and magazines, and assessing the internet.

## 3. Results

### 3.1. Sample Characteristics

The socioeconomic and demographic characteristics of the samples surveyed in the baseline, mid- and post-campaign surveys are presented in Table 2. There were statistically significant differences between the samples in at least two of the three survey rounds on the following variables: age, education, employment status, intake of fruits and vegetables, vigorous physical activity, body mass index, self-reported health, frequency of watching television, reading the newspaper, and accessing the internet. However, the sample characteristics between surveys did not differ in terms of gender, parental status, socioeconomic status and frequency of listening to the radio.

### 3.2. Campaign Awareness

**Figure 4:**
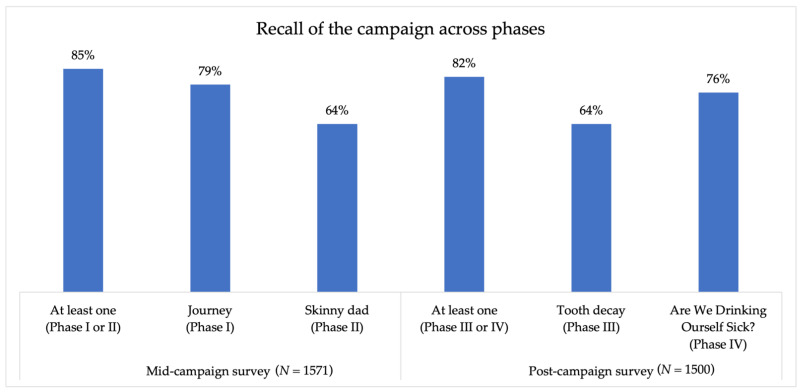
Recall of the “Are We Drinking Ourselves Sick?” campaign across phases.

Overall recall of the campaign was high in both the mid- and post-campaign surveys. In the mid-campaign survey, 85% of respondents were aware of the campaign, with 79% of respondents recalling the ad “Journey” and 64% recalling “Skinny Dad”. In the post-campaign survey, 82% of respondents were aware of the campaign, with 64% of respondents recalling the campaign ad “Tooth Decay” and 76% recalling one of the three ads in rotation in the series “Are We Drinking Ourselves Sick? Testimonies” (See Figure 4).

There were statistically significant sociodemographic differences between those who were campaign-aware and -unaware. In both the mid- and post-campaign surveys, campaign awareness was higher among women than men; older rather than younger adults; and those who consumed more media (television, radio, and newspaper). In the mid-campaign survey, parents/caregivers of children under the age of 16 and those who accessed the internet more frequently were more likely to be campaign aware, whereas there was no statistically significant difference for respondents in the post-campaign survey. In the post-campaign survey, greater campaign awareness was reported by those who engaged in more frequent vigorous physical activity and reported better health (See Table 3).

### 3.3. Reactions to the Campaigns

Overall, campaign-aware respondents to both the mid- and post-campaign surveys reacted positively to the campaign (see Table 4). More than 90% of those who were campaign-aware agreed that the campaign ad(s) were believable and personally relevant and that they would like others to see them. Parents strongly felt that they would like their children to see the ad(s) (99% mid-campaign, 95% post-campaign). In addition, most respondents (≥74%) agreed that the ad(s) taught them something new and made them stop and think. Moreover, the campaign ad(s) made respondents feel concerned about the effects of sugary drinks on their health (88% mid-campaign, 85% post-campaign), feel motivated to reduce their consumption (85% mid-campaign, 83% post-campaign), and made parents motivated to reduce their children’s consumption (87% mid-campaign, 85% post-campaign). Finally, the ads made respondents more supportive of government action to reduce sugary drink consumption (89% mid-campaign, 86% post-campaign).

### 3.4. Knowledge and Attitudes

#### 3.4.1. Changes from Baseline to Mid- and Post-Campaign Surveys

Overall, most respondents across survey waves (≥77%) knew that sugary drinks are a risk factor for diabetes, but there was a reduction in knowledge of this association from the baseline to post-campaign period (adjusted odds ratio or AOR = 0.62, *p* < 0.001). There was also a reduction from the baseline to post-campaign period in understanding of the association between sugary drinks and overweight and obesity (AOR = 0.58, *p* < 0.001); heart disease (AOR = 0.79, *p* = 0.003); and asthma (AOR = 0.69, *p* < 0.001). On the other hand, from the baseline to mid-campaign period, there was an increase in knowledge of the association between sugary drinks and asthma (AOR = 1.26, *p* = 0.012); hypertension (AOR = 1.22, *p* = 0.009); cancer (AOR = 1.36, *p* < 0.001); and early death (AOR = 1.37, *p* < 0.001).

Overall, most Jamaicans agreed that sugary drinks contribute to poor nutrition and to obesity in the country (≥80%), and thus are a cause for concern. Compared to the baseline, respondents to the mid-campaign survey were more likely to believe that sugary drinks are a major contributor to the obesity problem in Jamaica (AOR = 1.30, *p* = 0.015). On the other hand, respondents to the post-campaign survey were more likely than those in the baseline to believe that too much sugar would not affect their health if they exercised regularly (AOR = 1.21, *p* = 0.016), and were less likely to believe that Jamaicans are not aware of the health harms of sugary drinks (AOR = 0.79, *p* = 0.007) and that people important to them do not want them to consume sugary drinks (AOR = 0.77, *p* = 0.009).

#### 3.4.2. Impact of Campaign Awareness within the Mid-and Post-Campaign Periods

Table 5 describes the independent association between campaign awareness and knowledge, and it suggests that that campaign awareness may have prevented the backsliding of knowledge on the health risks of sugary drinks that was observed for those who were unaware.

Respondents to the post-campaign survey who were campaign aware were significantly more likely than those who were unaware to know that sugary drink consumption increases the risk of diabetes (AOR = 1.45, *p* = 0.019), overweight and obesity (AOR = 1.65, *p* = 0.001), and heart disease (AOR = 1.44, *p* = 0.011).

Campaign awareness was associated with increased concerns about the risks of sugary beverages and less accepting social norms around their use. Campaign-aware respondents were more likely than those who were unaware to believe that sugary drinks are a major contributor to the obesity problem in the country (AOR = 1.40, *p* = 0.050) and that they are a major source of unnecessary sugar in one’s diet (AOR = 1.59, *p* = 0.010). In addition, campaign awareness was associated with an increased belief among post-campaign respondents that social norms are unsupportive of children drinking sugary drinks (AOR = 1.55, *p* = 0.008) and increased concerns about the effects of drinking sugary drinks on their own health (AOR = 1.45, *p* = 0.010).

### 3.5. Support for Government Action

#### 3.5.1. Changes from Baseline to the Mid- and Post-Campaign Surveys

Overall support for prompt government action to address the obesity epidemic in Jamaica was high across all survey waves (≥90%) (see Table 6). Compared to the baseline, mid- and post-campaign survey respondents were more likely to support government efforts to pass and enforce policies to discourage drinking sugary drinks and consuming non-essential foods (mid-campaign: AOR = 1.22, *p* = 0.030; post-campaign: AOR = 1.20, *p* = 0.040).

Overall, more than half of respondents across all survey waves supported taxes on sugary drinks (≥55%) to reduce obesity in Jamaica. This support increased substantially when the policy included funneling revenue into public programs to improve health (≥71%). Respondents to both the mid- and post-campaign surveys were more likely to support taxing sugary drinks than those in the baseline (mid-campaign: AOR = 1.29, *p* < 0.001; post-campaign: AOR = 1.17, *p* = 0.050). They were also more likely than those in the baseline to support a tax on unhealthy foods (mid-campaign: AOR = 1.24, *p* < 0.001; post-campaign: AOR = 1.24, *p* = 0.010) and a tax on sugary drinks where the revenue goes to public health programs (mid-campaign: AOR = 1.42, *p* < 0.001; post-campaign: AOR = 1.40, *p* < 0.001).

Public support was highest when it came to requiring the provision of healthy foods and drinks in schools and increasing children’s access (≥93%), but though still high, was lower when it came to restricting the sale or provision of sugary drinks or unhealthy foods in schools (≤72%). Compared to the baseline, mid- and post-campaign survey respondents were more likely to support increasing children’s access to healthy foods and drinks (mid-campaign: AOR = 1.69, *p* < 0.001; post-campaign: AOR = 1.49, *p* = 0.20). Post-campaign respondents were also more likely than those in the baseline to support restrictions on the sale and/or provision of sugary drinks and unhealthy foods in schools (AOR = 1.28, *p* = 0.010).

#### 3.5.2. Impact of Campaign Awareness within the Mid-and Post-Campaign Periods

For both mid- and post-campaign respondents, campaign awareness was associated with increased support for a tax on sugary drinks (mid-campaign: AOR = 1.43, *p* = 0.019; post-campaign: AOR = 1.46, *p* = 0.010), and unhealthy foods (mid-campaign: AOR = 1.68, *p* < 0.001; post-campaign: AOR = 1.42, *p* = 0.010). Those who were campaign aware in the post-campaign period were also more likely than those who were unaware to support taxes on drinks (AOR = 1.39, *p* = 0.040), and unhealthy food (AOR = 1.53, *p* = 0.010) when it was specified that revenue would be used for public health programs. Respondents to the post-campaign survey who were campaign aware were significantly more supportive of restricting the sale or provision of sugary drinks and unhealthy foods in schools than those who were unaware (AOR = 1.55, *p* = 0.010).

## 4. Discussion

The “Are We Drinking Ourselves Sick?” campaign was successful in achieving high recall and it was well-received. More than 80% of those surveyed recalled the campaign, and the vast majority found the campaign to be believable, relevant, and motivating in reducing their own and their children’s’ consumption of sugary drinks. Importantly, the campaign was reported to be motivating of support for government action to reduce sugary drinks consumption.

Our study showed attenuation in knowledge on some measures over the survey period. Even as the majority continued to recognize the harms of sugary drinks, our surveys picked up a decline between the baseline and post-campaign period in knowledge of sugary drinks being a major contributor to obesity in Jamaica; that they can increase the risk of diabetes, overweight and obesity; heart disease; and an increasing belief that exercise can neutralize the ill-effects of sugary drinks. There may have been a number of possible explanations for this observed attenuation in population-wide knowledge. As described in Table 1, phases III and IV of the “Are We Drinking Ourselves Sick” campaign focused more on policymakers and were also mounted with a lower media budget and ran for a shorter duration and may not have had the population reach of the earlier two phases. Finally, other communication campaigns that ran concurrently may have diluted the messaging in the “Are We Drinking Ourselves Sick” campaign. This includes campaigns promoting physical activity that ran during this time, which may have also contributed to public uncertainty over the relative contributions of diet versus physical activity in the prevention of overweight and obesity and diet-related illnesses. In addition, campaigns against policies to reduce sugary drink consumption may have contributed to the observed attenuation in public knowledge [43,44,45,46,47].

The media campaign, however, had a protective effect from attenuation on knowledge, attitudes, and social norms toward sugary drinks. In the post-campaign period, those who were campaign aware were much more likely than those who were unaware to know that sugary drinks are a risk factor for overweight and obesity and that they are a major source of unnecessary sugar in one’s diet. In addition, they were more likely to feel that social norms in Jamaica do not support children’s consumption of sugary drinks and to have increased concern about the effects of these beverages on their own health. These findings suggest that media campaigns can play an important role in maintaining the health agenda for diet-related policies.

Our findings show that support for policy action was high and increased across survey waves: overall, nine in ten Jamaicans want action on obesity. Specifically, more than half of the respondents supported implementing taxes on sugary drinks. In line with other studies, this support increased substantially when the policy specifically mentioned funneling revenue into public programs to improve health [48,49,50]. These findings were consistent with those from a study in South Africa based on a similar campaign, albeit in a different cultural setting [42]. In addition, more than two-thirds of Jamaicans supported restricting the sale or provision of sugary drinks in schools. Campaign awareness was independently associated with this increased support. Notably, campaign awareness was associated with increased support of taxes on sugary beverages irrespective of whether it was specified how funds would be used. For respondents to the mid-campaign survey, in particular, campaign awareness increased their support of a tax on sugary drinks and unhealthy foods but did not affect their support for taxes if funds were earmarked for public health programs. This suggests that the campaigns were successful in conveying the immediate health need for the tax and in garnering support based on the perceived health benefits of the policy. For those in the post-campaign period, awareness of the campaign also increased support for restricting the sale of unhealthy foods and drinks in schools.

Our study has several important limitations that are worth noting. First, we did not measure concurrent activities beyond the campaign, such as direct advocacy and lobbying efforts, and policy changes such as the restriction of sugary drinks in school, which may also have contributed to policy support gains observed between survey waves. Our study makes an important contribution in demonstrating the independent role of the media campaign in building public support for policies on sugary drinks, but future research may consider the complementary and multiplicative effects of contiguous interventions in maintaining such support. In addition, we did not track the extent of food and beverage industry-sponsored marketing in favor of sugary drinks consumption during this period, which likely also affected the observed pattern of findings, including the decline in knowledge about sugary drinks that we observed in the post-campaign period [51,52,53]. Such an analysis would have more clearly demonstrated the contributory role of the media campaign described in this paper, and as such, this study may represent an underrepresentation of its role.

This study supports the importance of communication to maintain a public understanding of harms in a crowded media environment and increase support for policies to address sugary drinks consumption. It adds to the literature on how mass media campaigns can be used to boost public support for health policies, particularly those that are more contentious and generally are perceived to have lower levels of approval such as fiscal policies to tax unhealthy products and policies that restrict the sale of unhealthy products [12,20,22]. These types of policy proposals may especially benefit from the use of a mass media campaign to make the public health case for such measures.

## 5. Conclusions

This study demonstrates how the “Are We Drinking Ourselves Sick?” mass media campaign in Jamaica contributed to higher levels of support for a tax on sugary drinks and restrictions on the provision of these products in schools. The campaigns’ ability to increase support for a tax on sugary drinks, which is an effective but more contentious policy, is especially noteworthy. In addition, it is evident that the campaign prevented an erosion of knowledge about sugary drinks’ contribution to overweight, obesity, and diet-related diseases. Thus, mass media campaigns serve as a strong tool for public health advocates and governments to maintain public concern over diet-related health issues and build support for policies to address them.

## Figures and Tables

**Figure 1:**
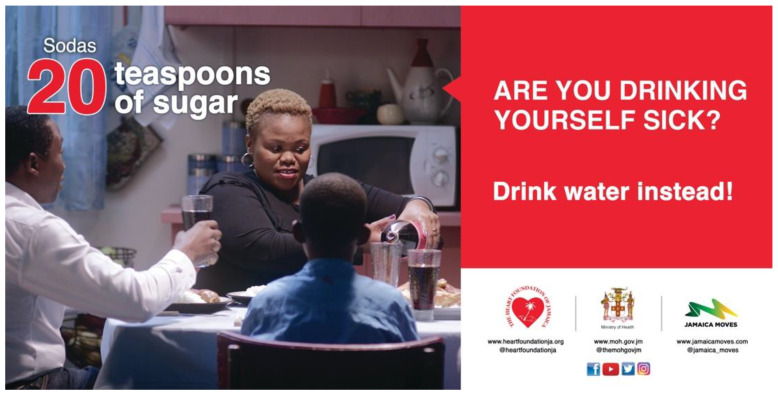
Superboard ad, Jamaica, 2017—“Are You Drinking Yourself Sick?” (Phase I).

**Figure 2:**
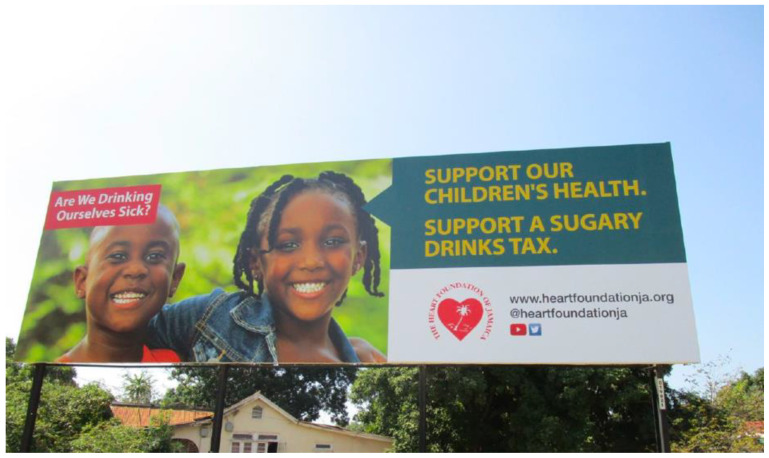
Billboard in St. Andrew, Jamaica, 2019—“Are We Drinking Ourselves Sick?” (Phase IV).

**Table 2:** Demographics characteristics of survey respondents in baseline, mid-campaign, and post-campaign.

	Baseline	Mid-Campaign	Post-Campaign	*χ*^2^ *p*-Value ^a^
	(*n* = 1430)	(*n* = 1571)	(*n* = 1500)	
	A	B	C	
Average age (years)	33	33	34 A,B	<0.001
Women (%)	52	50	50	0.557
Parent/primary caregiver to children under the age of 16 (% yes)	46	49	51	0.055
Education (%)				<0.001
No formal schooling	22 C	19 C	13	
Below high school	76	80 A	84 A,B	
High school and above	2 B	0.7	4 A,B	
Employment status (%)				<0.001
Unemployed	7	21 A,C	17 A	
Employed	76 B	72	76	
Student	17 B,C	7	7	
Socioeconomic status (%)				0.059
Upper and upper middle socioeconomic status (ABC1)	16	13	15	
Middle socioeconomic status (C2)	26	24	25	
Low and low socioeconomic status (DE)	58	63 A	61	
Fruits and vegetables intake in the last seven days (%)				<0.001
Two or less than two times	69	71	84 A,B	
More than two times	31 C	30 C	17	
Average vigorous physical activity in the last seven days (days)	1.7	2.1 A,C	1.7	<0.001
Body Mass Index (%)				0.046
Underweight	5	3	7 B	
Normal weight	39	41	37	
Overweight	30	29	32	
Obese	26	27	24	
Self-rated health status (%)				<0.001
Poor/fair	47 B,C	36	34	
Good	28	31	33 A	
Very good/excellent	25	32 A	33 A	
Frequency of watching television on a typical week (%)				<0.001
Never	14 C	11 C	6	
Three or less than three times	32	35	38 A	
More than three times	54	54	57	
Frequency of listening to the radio on a typical week (%)				0.091
Never	23	24	23	
Three or less than three times	31	34	35	
More than three times	46 B	42	42	
Frequency of reading newspaper/magazines/periodicals in a typical week (%)				0.007
Never	35	38	42 A	
Three or less than three times	53 C	51	48	
More than three times	12	11	11	
Frequency of access to the internet (%)				0.001
Never	13 C	13 C	9	
Once or more than once a week	16	16	14	
Once or more than once a day	71	71	77 A,B	

Results are based on two-sided tests. For each significant pair, the key of the category with the smaller column proportion appears in the category with the larger column proportion. ^a^ Tests are adjusted for all pairwise comparisons within a row of each innermost subtable using the Bonferroni correction. Significance level for upper case letters (A,B,C): *p* < 0.05. *χ*^2^, chi-square.

**Table 3:** Demographic characteristics of campaign-aware and campaign-unaware respondents in the mid-campaign survey and post-campaign survey period.

	Mid-Campaign			Post-Campaign		
	Unaware	Aware	*χ*^2^ *p*-Value	Unaware	Aware	*χ*^2^ *p*-Value
	(*n* = 239)	(*n* = 1332)		(*n* = 271)	(*n* = 1229)	
Average age (years)	32	33	0.013	32	35	<0.001
Gender (%)			<0.001			0.004
Men	19	81		21	79	
Women	11	89		15	85	
Parent/primary caregiver to children under the age of 16 (% yes)			0.014			0.88
Yes	13	87		18	82	
No	17	83		18	82	
Education (%)			0.309			0.595
No formal schooling	16	84		21	79	
Below high school	15	85		18	82	
High school and above	0	100		17	83	
Employment status (%)			0.226			0.207
Unemployed	12	88		15	85	
Employed	16	84		19	81	
Student	17	83		14	86	
Socioeconomic status (%)			0.874			0.171
Upper and upper middle socioeconomic status (ABC1)	16	84		17	83	
Middle socioeconomic status (C2)	15	86		15	85	
Low and low socioeconomic status (DE)	15	85		20	81	
Fruits and vegetables intake in the last seven days (%)			0.405			0.219
Two or less than two times a day	15	85		19	81	
Three or more than three times a day	16	84		15	85	
Average vigorous physical activity in the last seven days (days)	2	2	0.296	1	2	0.002
Body Mass Index (%)			0.613			0.143
Underweight	10	90		22	78	
Normal weight	14	86		18	82	
Overweight	16	84		11	89	
Obese	12	88		15	85	
Self-reported heath (%)			0.072			<0.001
Poor/fair	18	82		23	78	
Good	13	87		19	81	
Very good/excellent	14	86		13	87	
Frequency of watching television in a typical week (%)			<0.001			<0.001
Never	42	58		41	59	
Three or less than three times	17	83		23	77	
More than three times	9	91		13	88	
Frequency of listening to the radio in a typical week (%)			<0.001			<0.001
Never	23	77		26	74	
Three or less than three times a week	15	85		20	81	
More than three times a week	11	89		12	88	
Frequency of reading magazines/periodicals/newspapers in a typical week (%)			<0.001			<0.001
Never	20	80		24	76	
Three or less than three times a week	12	88		14	86	
More than three times a week	12	88		14	86	
Accessing the internet (%)			0.048			0.979
Never	16	84		18	82	
Once or more than once a week	10	90		18	82	
Once or more than once a day	16	84		18	82	

Significant at *p* < 0.05. *χ*^2^ chi-square.

**Table 4:** Reactions to the campaign materials among those who recalled it through any medium.

Reactions to the Campaigns	Mid-Campaign Aware (*n* = 1332)	Post-Campaign Aware (*n* = 1229)
	%	%
Agreed that the campaign...		
Was believable	96	92
Was relevant to me	93	91
Taught me something new	78	74
Made me stop and think	84	80
Made me feel uncomfortable	33	35
Made me feel concerned about the impact of sugary drinks on my health	88	85
Made me motivated to reduce drinking sugary drinks	85	83
Made me want to discuss the ad with others	78	75
Made me more supportive of government action to reduce sugary drink consumption in my country	89	86
I would like others to see this ad	98	95
This ad provides a public service/it is in the public’s interest to watch	98	95
This ad made me motivated to drink more water	90	––
This ad motivated me to read food labels	84	––
I would like children to see the ad [parents or primary caregivers only; mid-campaign (*n*) = 772; post campaign (*n*) = 759]	99	95
Made me motivated to reduce my child’s drinking of sugary drinks [parents or primary caregivers only; mid-campaign (*n*) = 772; post campaign (*n*) = 759]	87	85
Made me feel concerned about the impact of sugary drinks on my children’s health [parents or primary caregivers only; mid-campaign (*n*) = 772; post campaign (*n*) = 759]	––	86
Discussed the campaign with…		
Family	29	27
Friends	12	9
Colleagues	2	2
Others	3	3
Likelihood of reducing the number of sugary drinks consumed as a result of seeing the campaign (likely)	65	59
Likelihood of reducing the number of sugary drinks consumed by your children as a result of seeing the campaign (likely)	76	68
Support for ads like this one on the health effects of consumption of nonessential food and sugary drinks (support)	95	97

–– The questions were not asked in a particular survey. *n*, sample size.

**Table 5:** Knowledge and attitude towards obesity and sugary drinks among respondents in the pre-campaign and post-campaign periods including a comparison of post-campaign respondents who were aware and unaware of the campaign.

	Baseline Survey	Mid-Campaign Survey	Post-Campaign Survey	Mid-Campaign Survey		Post-Campaign Survey		Mid-Campaign Survey			Post-Campaign Survey			
								Unaware	Aware		Unaware	Aware		
	(*n* = 1430)	(*n* = 1571)	(*n* = 1500)	Ref: Baseline Survey				(*n* = 239)	(*n* = 1332)		(*n* = 271)	(*n* = 1229)		
	A	B	C					D	E		F	G		
	%	%	%	Adj. OR ^ (95% CI)		Adj. OR ^ (95% CI)		%	%	Adj. OR ^ (95% CI)	%	%	Adj. OR ^ (95% CI)	
**Knowledge About Sugary Drinks**														
To the best of your knowledge, does drinking sugary drinks increase the risk of…? (somewhat/greatly)														
Diabetes	84 C	84 C	77	1.018 (0.83, 1.25)		0.62 ** (0.51, 0.76)	↓	81	85	1.26 (0.85, 1.86)	71	78 F	1.45 * (1.06, 1.99)	↑
Overweight or obesity	78 C	79 C	67	1.1 (0.92, 1.31)		0.58 * (0.49, 0.69)	**↓**	74	79 D	1.2 (0.85, 1.70)	56	69 F	1.65 ** (1.24, 2.19)	↑
Heart disease, including heart attacks	58 C	61 C	52	1.14 (0.98, 1.32)		0.79 * (0.68, 0.92)	**↓**	56	62	1.24 (0.92, 1.68)	44	53 F	1.44 * (1.09, 1.90)	↑
Asthma	19 C	23 A,C	14	1.26 * (1.05, 1.51)	**↑**	0.69 * (0.56, 0.84)	**↓**	24	23	0.86 (0.61, 1.23)	13	14	1.01 (0.67, 1.52)	
Hypertension or high blood pressure	56	61 C	53	1.22 * (1.05, 1.42)	**↑**	0.897 (0.77, 1.06)		57	61	1.17 (0.86, 1.59)	47	54 F	1.28 (0.97, 1.69)	
Cancer	33	40 A,C	31	1.36 ** (1.16, 1.58)	**↑**	0.972 (0.83, 1.15)		39	40	0.99 (0.73, 1.35)	26	33 F	1.37 (1.00, 1.88)	
Dental problems	62	65 C	61	1.13 (0.97, 1.31)		0.955 (0.82, 1.12)		63	66	1.19 (0.88, 1.62)	56	62	1.19 (0.90, 1.58)	
Early death (premature death)	49	56 A,C	49	1.37 ** (1.18, 1.58)	↑	1.01 (0.87, 1.18)		54	57	1.17 (0.87, 1.58)	44	50	1.2 (0.90, 1.58)	
**Attitudes Towards Sugary Drinks**														
Sugary drinks are a major contributor to the obesity problem in Jamaica.	84	87	80	1.3 * (1.05, 1.60)	↑	0.823 (0.67, 1.01)		83	87	1.32 (0.88, 1.98)	76	81 F	1.4 * (1.00, 1.95)	↑
Sugary drinks are the major source of unnecessary sugars in a person’s daily diet.	84	86	84	1.17 (0.99, 1.44)		0.928 (0.76, 1.14)		86	86	0.9 (0.58, 1.40)	79	85 F	1.59 * (1.12, 2.25)	↑
Too much sugar can cause severe health problems	96	96	95	0.92 (0.63, 1.35)		0.738 (0.51, 1.08)		94	96	1.25 (0.63, 2.50)	93	95	1.4 (0.79, 2.50)	
Concerned about the effect of drinking sugary drinks on your health? (concerned)	64	66	63	1.11 (0.95, 1.30)		0.981 (0.84, 1.15)		63	67	1.04 (0.77, 1.42)	53	65 F	1.45 * (1.09, 1.92)	↑
Concerned about the effect of drinking sugary drinks on your children’s health? (concerned)	78	78	79	1.03 (0.79, 1.33)		1.133 (0.86, 1.49)		75	78	1.19 (0.70, 2.00)	80	79	0.75 (0.43, 1.29)	
As long as I exercise regularly, too much sugar will not harm my health.	39	41	46 A	1.03 (0.89, 1.20)		1.21 * (1.04, 1.41)	↑	45	41	1 (0.73, 1.35)	44	46	1.11 (0.84, 1.47)	
**Social Norms Around Sugary Drinks**														
There are fewer and fewer occasions in which I feel comfortable drinking sugary drinks.	70	68	73 B	0.93 (0.79, 1.09)		1.11 (0.94, 1.31)		64	69	1.09 (0.79, 1.50)	69	74	1.22 (0.90, 1.65)	
People in my country are unaware of the health harms of sugary drinks.	74 C	72	69	0.9 (0.76, 1.06)		0.79 * (0.67, 0.94)	↓	72	71	1.03 (0.74, 1.43)	68	70	1.08 (0.80, 1.45)	
The people important to me believe that I should avoid or stop drinking sugary drinks.	83	81	80	0.86 (0.71, 1.04)		0.77 * (0.63, 0.94)	↓	81	81	0.84 (0.570, 1.23)	78	80	1.17 (0.83, 1.64)	
People important to me disapprove of children drinking sugary drinks frequently.	77	77	79	0.99 (0.83, 1.18)		1.077 (0.89, 1.29)		75	77	1.01 (0.71, 1.43)	73	80 F	1.55 * (1.12, 2.15)	↑

Abbreviations: Ref, reference category; LL, lower limit; UL, upper limit; OR, odds ratio; CI, confidence interval. Significance level for upper case letters (A, B, C, D, E, F, G): *p <* 0.05. * Significant at *p* < 0.05; ** significant at *p* < 0.01. ^ Covariates adjusted for include age, gender, parental status, self-reported health, socioeconomic status, vigorous physical activity in the last seven days, frequency of watching television, listening to the radio, and reading the newspaper in the last seven days, access to internet, and fruit and vegetable intake. A significant adjusted odds ratio indicates that even after confounding factors have been taken into account, the odds of the “aware” group’s reported intentions/behaviors are significantly different from the odds of the “unaware” group’s reported intentions/behaviors. ↑ AOR significantly increased in comparison to the ref category. ↓ AOR significantly decreased in comparison to the ref category.

**Table 6:** Support for the government’s efforts regarding obesity and sugary drinks among respondents in the pre-campaign, post-campaign I, and post-campaign II periods including a comparison of evaluation survey’s respondents who were aware and unaware of the campaign.

	Baseline Survey	Mid-Campaign Survey	Post-Campaign Survey	Mid-Campaign Survey		Post-Campaign Survey		Mid-Campaign Survey				Post-Campaign Survey			
								Unaware	Aware	Adj. OR ^ (95% CI)		Unaware	Aware	Adj. OR ^ (95% CI)	
	(*n* = 1430)	(*n* = 1571)	(*n* = 1500)	Ref: Baseline Survey				(*n* = 239)	(*n* = 1332)			(*n* = 271)	(*n* = 1229)		
	A	B	C	Adj. OR ^ (95% CI)		Adj. OR ^ (95% CI)		D	E			F	G		
	%	%	%					%	%			%	%		
To what extent do you agree or disagree with the following statements? (% agree)															
My government must act quickly to implement policies to address the problem of obesity in my country.	90	91	90	1.12 (0.88, 1.44)		0.97 (0.76, 1.25)		88	91	1.28 (0.79, 2.05)		87	91	1.39 (0.91, 2.11)	
The government should pass and enforce policies that discourage drinking sugary drinks and eating non-essential foods that are high in sugar, salt, and fat.	72	77 A	77 A	1.22 * (1.03, 1.44)	↑	1.2 * (1.01, 1.43)	↑	72	78 D	1.22 (0.87, 1.71)		72	78 F	1.29 (0.94, 1.78)	
How strongly would you agree with or disagree with each of the following policies as a way of reducing obesity in Jamaica?															
Taxes on sugary drinks.	55	61 A	59	1.29 * (1.11, 1.50)	↑	1.17 * (1.00, 1.36)	↑	52	62 D	1.43 * (1.06, 1.93)	↑	51	61 F	1.46 * (1.11, 1.93)	↑
Taxes on unhealthy foods that are high in sugar, salt or fats, such as ice-creams, chips, burgers, and buns.	54	59 A	59 A	1.24 ** (1.07, 1.44)	↑	1.24 * (1.07, 1.45)	↑	46	61	1.68 * (1.24, 2.26)	↑	51	61 F	1.42 * (1.08, 1.88)	↑
Taxes on sugary drinks if the money collected is invested in public programs to improve health.	71	77 A	77 A	1.42 ** (1.20, 1.69)	↑	1.4 ** (1.18, 1.67)	↑	74	78	1.18 (0.84, 1.67)	↑	72	79 F	1.39 * (1.02, 1.91)	↑
Taxes on unhealthy foods that are high in sugar, salt or fats, such as ice-creams, chips, burgers, and buns if the money collected is invested in public programs to improve health.	76	76	74	1 (0.84, 1.19)		0.86 (0.72, 1.03)		70	77 D	1.23 (0.88, 1.72)		66	75 F	1.53 * (1.13, 2.07)	↑
To what extent do you agree or disagree with the following statements? (agree)															
I intend to support government efforts to increase children’s access to healthy foods and drinks.	93	96 A	96 A	1.69 ** (1.21, 2.37)	↑	1.49 * (1.06, 2.11)	↑	93	97 D	1.63 (0.85, 3.12)		94	96	1.29 (0.69, 2.42)	
I support restriction on the sale and/or provision of sugary drinks and unhealthy foods in schools.	72	75	77 A	1.149 (0.97, 1.36)		1.28 * (1.07, 1.52)	↑	73	75	0.95 (0.68, 1.34)		70	78 F	1.55 * (1.14, 2.11)	↑
I support requiring the provision of healthy food and beverages in schools.	95	95	96 A	0.76 (0.54, 1.09)		1.01 (0.68, 1.49)		93	95	1.35 (0.75, 2.44)		96	96	1.21 (0.61, 2.38)	

Abbreviations: Ref, reference category; LL, lower limit; UL, upper limit; OR, odds ratio; CI, confidence interval. Significance level for upper case letters (A, B, C, D, E, F, G): *p <* 0.05. * Significant at *p* < 0.05; ** significant at *p* < 0.01. ^ Covariates adjusted for include age, gender, parental status, self-reported health, socioeconomic status, vigorous physical activity in the last seven days, frequency of watching television, listening to the radio, and reading the newspaper in the last seven days, access to internet, and fruit and vegetable intake. A significant adjusted odds ratio indicates that even after confounding factors have been taken into account, the odds of the “aware” group’s reported intentions/behaviors are significantly different from the odds of the “unaware” group’s reported intentions/behaviors. ––The question was not asked for the particular survey. ↑ AOR significantly increased in comparison to the ref category.

## Data Availability

Additional data are available from the corresponding author on reasonable request.
